# Effect of BRCA1 on the Concurrent Chemoradiotherapy Resistance of Cervical Squamous Cell Carcinoma Based on Transcriptome Sequencing Analysis

**DOI:** 10.1155/2020/3598417

**Published:** 2020-06-21

**Authors:** Xin Wen, Shui Liu, Manhua Cui

**Affiliations:** ^1^Department of Gynecology and Obstetrics, The Second Hospital of Jilin University, Changchun, Jilin, China; ^2^The Second Hospital of Jilin University, Changchun, Jilin, China

## Abstract

**Background:**

Cervical squamous cell carcinoma (CSCC) is the main pathological type of cervical cancer, accounting for 80%–85% of cervical cancer. Owing to concurrent chemoradiotherapy (CCRT) resistance in a subset of CSCC patients, the treatment response is often unsatisfactory. Identifying predictors and therapeutic targets related to cisplatin-based CCRT resistance in CSCC is critical.

**Methods:**

We reanalyzed GSE56363, an mRNA dataset from the GEO database with 21 patients with locally advanced CSCC, to identify differentially expressed genes (DEGs) related to CCRT resistance. The hub genes were screened from the protein-protein interaction network of DEGs using cytoHubba plug-in of Cytoscape software. Transcriptome sequencing technology was used to compare differential expression between SiHa cells overexpressing BRCA1 compared with control SiHa cells. Functional annotation for DEGs and gene set enrichment analysis (GSEA) was performed to identify DEG-enriched relative signaling pathways to examine the molecular mechanisms of BRCA1 in CCRT resistance of CSCC. qPCR was used to verify the expression of key genes in SiHa/DDP cells.

**Results:**

A total of 609 DEGs including 223 upregulated DEGs and 386 downregulated DEGs were identified between the complete response to CCRT (CR) and noncomplete response to CCRT (NCR) CSCC patients based on the GSE56363 dataset. Ten hub genes with the highest degrees were identified via the plug-in CytoHubba in Cytoscape: BRCA1, CDCA8, ASPM, CDC45, RAD51, HMMR, CENPF, EXO1, DTL, and ZWINT genes, and BRCA1 ranked first. Through transcriptome sequencing analysis based on GSE141558, 1344 DEGs were identified in BRCA1-overexpressing SiHa cells, including 824 upregulated DEGs and 520 downregulated DEGs. GSEA results showed that CCRT-resistance related signaling pathways, such as the JAK/STAT signaling pathway and the WNT signaling pathway, were differentially enriched in BRCA1-expressing SiHa cells. STAT1, STAT2, and CCND1 were screened as the differentially expressed target genes of BRCA1 and may correlate with resistance of CSCC. qPCR results showed that only STAT1 was significantly increased in SiHa cells with GV230-BRCA1 plasmid transfection.

**Conclusion:**

BRCA1 overexpression in SiHa cells may upregulate STAT1 to activate the JAK/STAT signaling pathway, suggesting a mechanism for enhanced CCRT resistance.

## 1. Introduction

Cervical cancer (CC) ranks fourth for both incidence and mortality among cancers in women worldwide, and there were an estimated 570,000 new cases and 311,000 deaths related to CC in 2018 [1]. Cervical squamous cell carcinoma (CSCC) is the main pathological type of CC, accounting for 80%–85% of all CC cases. Approximately two-thirds of CSCC patients are diagnosed at medium or advanced stage, and the standard treatment is a concurrent chemoradiotherapy (CCRT) regimen consisting of cisplatin-based chemotherapy, external beam radiotherapy, and brachytherapy [2]. However, owing to CCRT resistance in a subset of patients, the treatment response is often unsatisfactory. Therefore, identifying efficient predictors and therapeutic targets related to CCRT is critical to improve the prognosis of CSCC patients.

With the rise of bioinformatics and high-throughput sequencing technologies, cancer development mechanisms, drug resistance, and prognosis can be better explained at the genetic level [3, 4]. These findings will contribute to understanding individual differences and tumor heterogeneity in cancer patients. These technologies have been applied to investigate predictors and therapeutic targets of radiotherapy and chemotherapy resistance in many tumor types [5–7]. However, relatively few studies have examined CCRT resistance in CSCC [8, 9].

The BRCA1 tumor suppressor responds to DNA damage by participating in DNA repair, mRNA transcription, cell cycle regulation, protein ubiquitination, and other cellular pathways [10, 11]. The DNA repair ability of damaged cells improves with increased intracellular BRCA1 expression, resulting in drug resistance. Multiple studies have shown that the expression level of BRCA1 gene in cancers is negatively correlated with the sensitivity to platinum drugs [12–14] and sensitivity to radiotherapy [15–17].

In the current study, we screened the differentially expressed genes (DEGs) related to CCRT resistance of CSCC and found that BRCA1 overexpression enhanced CCRT resistance in CSCC. Subsequently, we performed transcriptome sequencing to explore the potential mechanisms by which BRCA1 may enhance resistance in CSCC. Our findings may provide theoretical support and new strategies for the diagnosis and treatment of CSCC patients with CCRT resistance.

## 2. Materials and Methods

### 2.1. Identification of DEGs Related to CCRT Resistance in CSCC Based on the GEO Dataset

The GSE56363 database of CSCC patients was downloaded from the NCBI-GEO database [18]. The CSCC patients were treated by CCRT, and cisplatin-based treatments were used as chemotherapeutics. Patients were divided into two groups: complete response to CCRT (CR) and noncomplete response to CCRT (NCR). The DEGs between NCR and CR patients were reanalyzed using limma package in R [19]. False discovery rate (FDR) < 0.05 and ∣LogFoldChange(FC) | >1 were considered as the cut-off criterion.

### 2.2. Gene Ontology (GO) Enrichment Analysis and Construction of the Protein-Protein Interaction Network

We performed GO enrichment for the DEGs related to CCRT resistance in CSCC by clusterProfiler package [20]. The STRING database (https://string-db.org/) is an online resource that provides known direct and indirect protein-protein interaction (PPI). We mapped the DEGs to STRING [21] to reveal the PPI information and visualized the PPI network using Cytoscape 3.7.2 [22]. The combined scores (interaction scores) > 0.4 were considered as significant. We then analyzed the PPI network to screen the hub genes using the cytoHubba plug-in of Cytoscape software [23].

We selected the top 10 DEGs with the highest degrees for further analysis. The receiver operating characteristic (ROC) curves of the hub genes were generated by the pROC package in R [24]. The violin plots of the hub gene expression were developed by the ggplot2 package in R [25]. Heat maps of the hub gene expression were developed by the pheatmap package in R [26].

### 2.3. Cells, Cell Culture, and Plasmid Transfection

The SiHa human CSCC cell line was purchased from Cell Bank of the Chinese Academy of Sciences, and SiHa/DDP cells were purchased from Fenghui Bio-Technology Limited. The cells were cultured in DMEM medium (Gibco, USA) supplemented with 10% fetal bovine serum (Sangon, China) and 1% antibiotic-antimitotic solution (Gibco). The GV230-BRCA1 and GV230 plasmids were purchased from GeneChem (Shanghai, China). SiHa cells were transfected using Lipofectamine 3000 reagent (Invitrogen, USA) following the manufacturer's protocol. Transfected cells were incubated for 48 h before further analysis.

### 2.4. Reverse Transcription and Quantitative Real-Time PCR

Total RNA was extracted using TRIzol (Sangon, Shanghai, China) according to the manufacturer's protocols. The quantity and quality of RNA were measured using the NanoDrop 2000 spectrophotometer (Thermo Scientific, USA). qRT-PCR was performed using EasyScript® First-Strand cDNA Synthesis SuperMix (TransGen Biotech, Beijing, China) and 2x SG Fast qPCR Master Mix (Sangon Biotech, China) with the Roche LightCycler480 PCR system. The 2^-*ΔΔ*CT^ method was used to calculate relative expression. The primer sequences are listed in Table 1. Experiments were performed in triplicate.

### 2.5. Transcriptome Sequencing of mRNAs and Differential Expression Analysis

Sequencing libraries were produced using the Hieff NGS™ MaxUp Dual-mode mRNA Library Prep Kit for Illumina (YEASEN, Shanghai, China) based on the instructions from the manufacturer. First, total RNA was quantified using the Qubit 2.0 RNA Assay Kit (Life, USA) to determine the amount of total RNA added to the library construction. Purification and fragmentation of mRNA were performed using the mRNA Capture Beads and Frag/Prime buffer. Synthesis and purification of double-stranded DNA were conducted using 1st Strand Synthesis Reaction Buffer, 2nd Strand Synthesis Buffer, and Hieff NGS™ DNA Selection Beads before PCR. Amplification and purification of the library were performed by PCR method with the 2x Super Canace™ High-Fidelity Mix, primer mix, and Hieff NGS™ DNA Selection Beads. PCR products were collected and assessed using the Qubit2.0 DNA Test Kit (Life). Transcriptome sequencing was performed by Sangon Biotech Co. Ltd. (Shanghai, China). DEGs were detected by the negative binomial distribution test using the DESeq R package [27]. FDR < 0.05 and ∣LogFC | >1 were the cut-off criterion. Each group sample for transcriptome sequencing contained three biological replicates.

### 2.6. euKaryotic Ortholog Groups (KOG) Functional Annotation and Gene Set Enrichment Analysis (GSEA)

KOG is the NCBI annotation system based on the direct homologous relationship of genes, in which Clusters of Orthologous Groups of proteins (COG) are targeted at prokaryotes for eukaryotes. KOG currently has 4852 classifications and can be combined with evolutionary relationships to classify homologous genes from different species into different ortholog clusters.

To explore potential mechanisms underlying the effect of BRCA1 in CCRT resistance in CSCC, GSEA was performed to detect whether a priori defined set of genes showed statistically significant differential expression between the BRCA1 overexpression group and negative control group using Broad Institute software [28]. Gene sets with a nominal *P* value < 0.05 and FDR < 0.25 were considered significantly enriched.

Transcriptional Regulatory Relationships Unraveled by Sentenced-based Text mining Version 2.0 database (TRRUST) (https://www.grnpedia.org/trrust/) is a database based on the literature to reflect the relationship between transcriptional regulations [29]. To screen the mechanisms of BRCA1 in resistance in CSCC, we searched the targets of BRCA1 using the TRRUST Version 2 database.

### 2.7. Statistical Analysis

Statistical analysis was performed using GraphPad Prism 7.0 (GraphPad Software Inc., USA). FDR controlling was used to correct *P* values with the Benjamini Hochberg algorithm implemented in R 3.5.1 suite (Lucent Technologies). FDR < 0.05 and *P* value < 0.05 were considered statistically significant.

## 3. Results

### 3.1. Processing of Microarray Data and Identification of DEGs from the GEO Dataset

**Figure 1:**
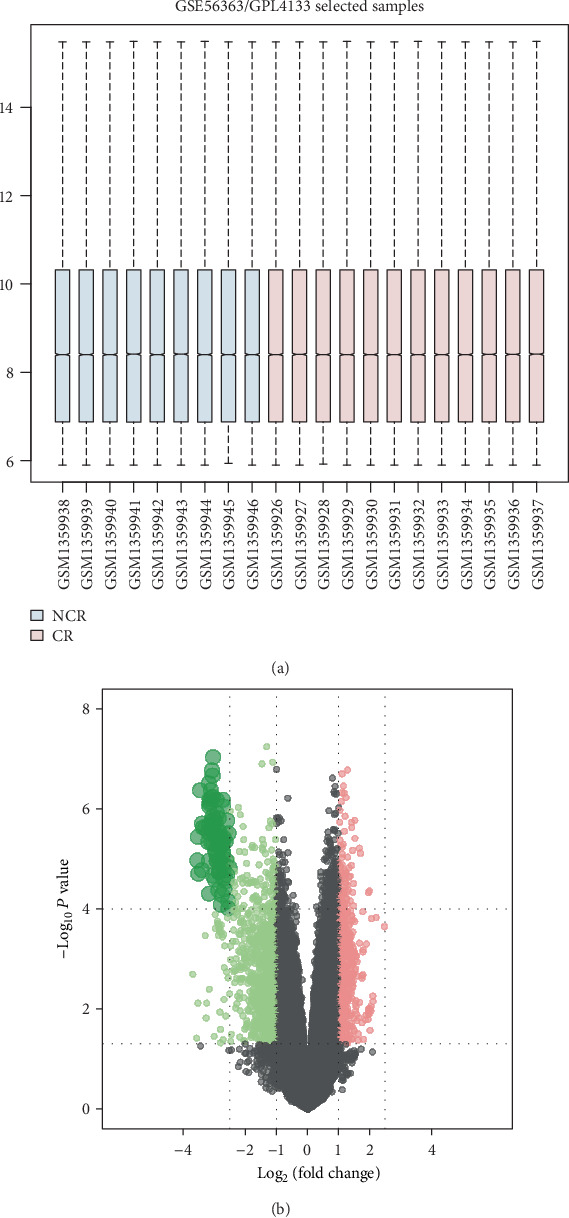
Processing of microarray data and identification of DEGs. (a) Boxplot of normalized expression values for GSE56363. The dotted line in the middle of each box represents the median of each sample, and its distribution among samples indicates the level of normalization of the data, with a straight line revealing a fair normalization level. (b) Volcano plot of DEGs based on GSE56363. The upregulated DEGs in NCR CSCC patients were signed in red, and downregulated DEGs were signed in green.

The GSE56363 dataset was normalized and the results are shown in Figure 1(a). A total of 21 CSCC patients at advanced stage including 9 NCR and 12 CR patients were analyzed. We obtained 609 DEGs including 223 upregulated DEGs and 386 downregulated DEGs in NCR patients compared with CR patients (Figure 1(b)).

### 3.2. GO Enrichment Analysis for DEGs

**Figure 2:**
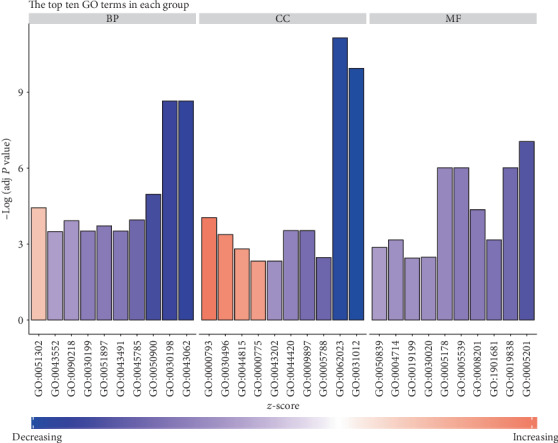
GO enrichment analysis of DEGs. The top 10 GO terms in each group were ranked by adjusted *P* value. The gradual color represents the *z*-score.

To obtain the function of the DEGs related to CCRT in CSCC, enrichment analysis of DEGs was performed using clusterProfiler in R. As shown in Table 2 and Figure 2, in the biological process group, the DEGs were most enriched in Response to virus. In the cellular component group, the DEGs were most enriched in the Integral component of lumenal side of endoplasmic reticulum membrane. In the molecular function group, the DEGs were most enriched in extracellular matrix structural constituent.

### 3.3. Construction of the PPI Network and Screening and Analysis of Hub Genes

**Figure 3:**
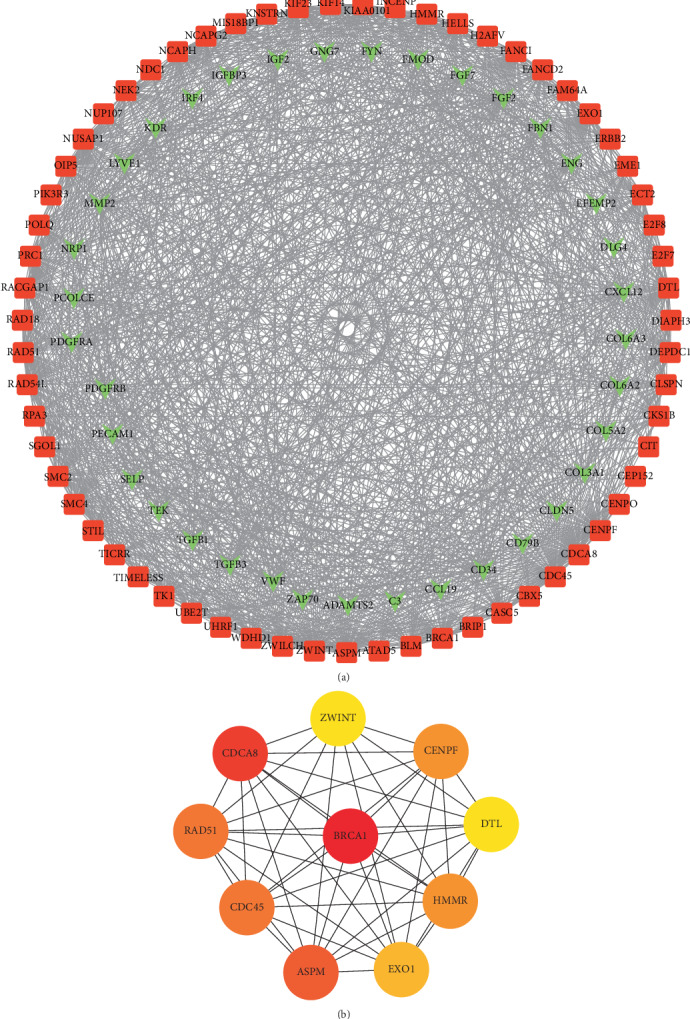
PPI networks. (a) PPI network of top 100 DEGs with the highest degrees. Red nodes denote upregulated genes, while green nodes denote downregulated genes. (b) PPI network of the hub genes. The gradual color represents the degree value.

**Figure 4:**
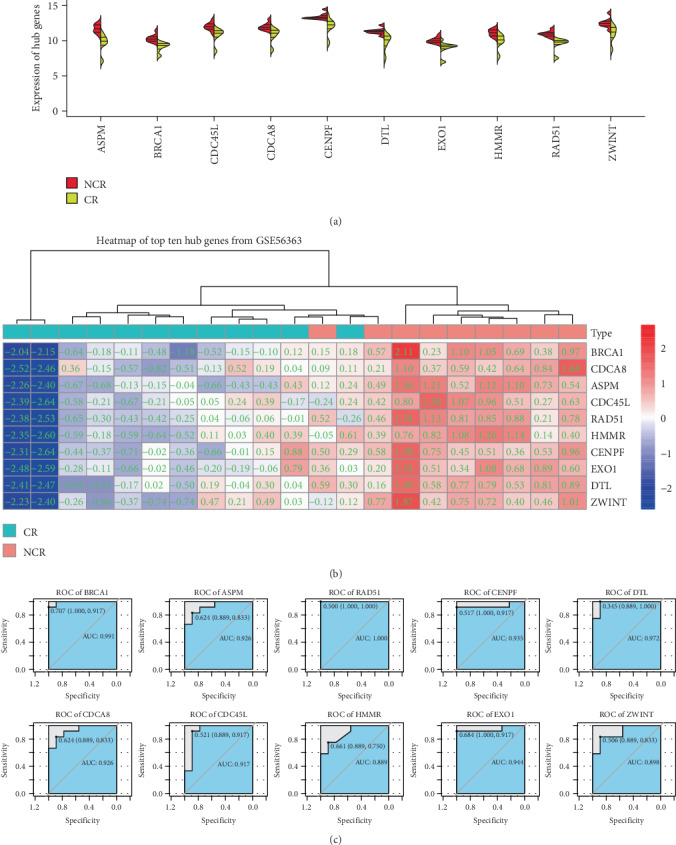
Comprehensive analysis of the hub genes. (a) Violin plots for different expressions of the hub genes between NCR and CR patients. (b) Heat maps of the hub genes in CSCC. (c) ROC curves of the hub genes sorted by AUC. NCR: noncomplete response; CR: complete response; ROC: receiver operating characteristic; AUC: area under the curve.

The PPI of the DEGs was collected by STRING and visualized in Cytoscape software. We evaluated the degree and betweenness centrality in the PPI network through the CytoHubba plug-in and identified the top 10 hub genes with the highest degrees: BRCA1, CDCA8, ASPM, CDC45, RAD51, HMMR, CENPF, EXO1, DTL, and ZWINT (Figure 3). Among them, BRCA1 has the highest degrees. The ROC curves of the hub genes all indicated favorable diagnostic value in CSCC patients with CCRT resistance (Figure 4).

### 3.4. Validation of BRCA1 Expression

**Figure 5:**
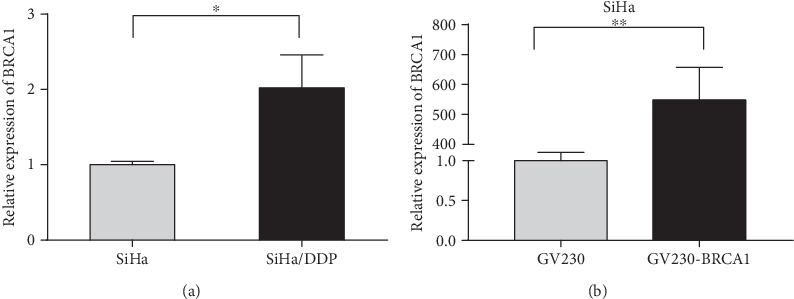
qRT-PCR. (a) The expression of BRCA1 by qRT-PCR between the SiHa/DDP group and the SiHa group. (b) The expression of BRCA1 in the SiHa cell with GV230-BRCA1 or GV230 transfection. ∗*P* < 0.05, ∗∗*P* < 0.01.

The qPCR results showed that the expression level of BRCA1 mRNA was significantly upregulated in SiHa/DDP cells compared with SiHa cells (Figure 5(a)). Furthermore, the expression level of BRCA1 was significantly increased in SiHa cell lines transfected with the GV230-BRCA1 plasmid (OV-BRCA1 group) compared with cells transfected with empty vector (NC group) (Figure 5(b)).

### 3.5. Identification and GO and KOG Enrichment of DEGs in SiHa Cells with BRCA1 Overexpression Plasmid Transfection

After removing reads with adapters, unknown nucleotides, and low-quality reads of sequencing, we acquired a total of 15.79 Gb clean data. The Q30 percentage of all samples was more than 90% (Table S1). The transcriptome sequencing data have been uploaded to the GEO database, and the accession number is GSE141558. We normalized the read count data using the FPKM method. Using FDR < 0.05 and ∣LogFC | >1 as the screening criterion in the process of gene identification, the transcriptome sequencing analysis identified 1344 DEGs between the OV-BRCA1 and NC groups, including 824 upregulated DEGs and 520 downregulated DEGs (Table S2).

**Figure 6:**
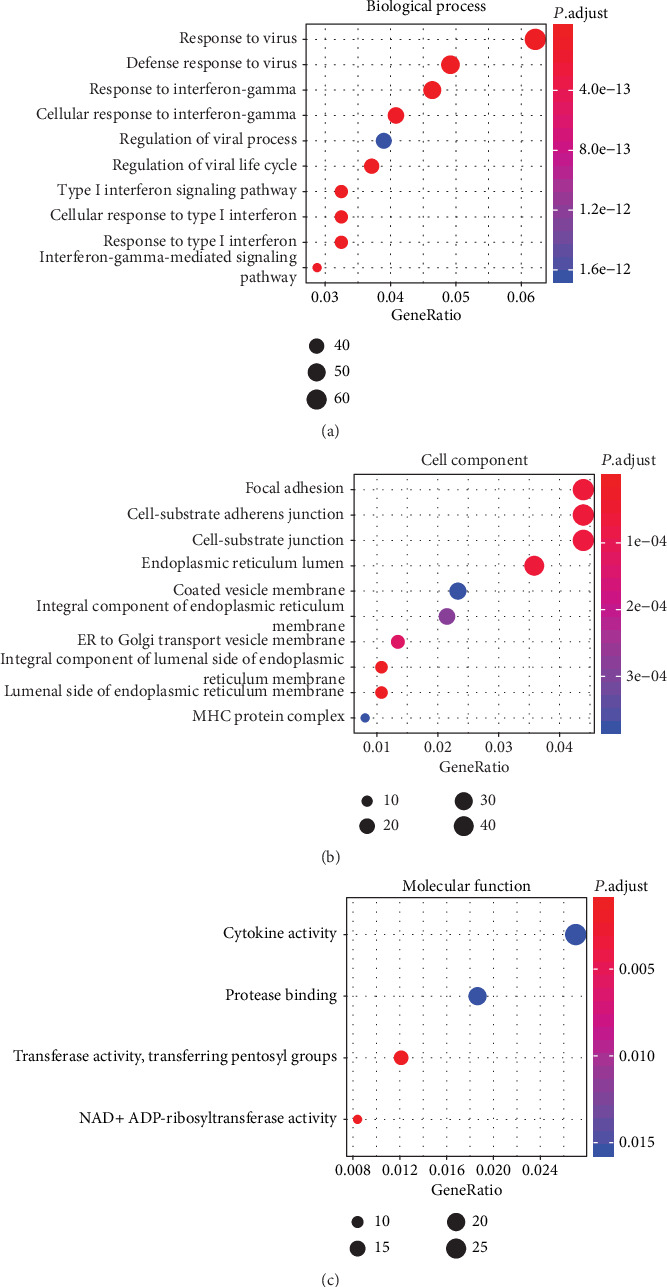
Dot plots for GO enrichment analysis of DEGs between the OV-BRCA1 and NC groups. (a) Dot plot of the top 10 GO terms in the biological process group. (b) Dot plot of the top 10 GO terms in the cell component group. (c) Dot plot of the top 4 GO terms in the molecular function group. The gradual color of the dots indicates the adjusted *P* value, and the size of the dots indicates the number of genes in the dot plots.

The enrichment analysis results showed that in the biological process group, the DEGs were most enriched in response to virus; in the cellular component group, the DEGs were most enriched in the nucleosome; and in the molecular function group, the DEGs were most enriched in transferase activity transferring pentosyl groups Table 3 and Figure 6).

**Figure 7:**
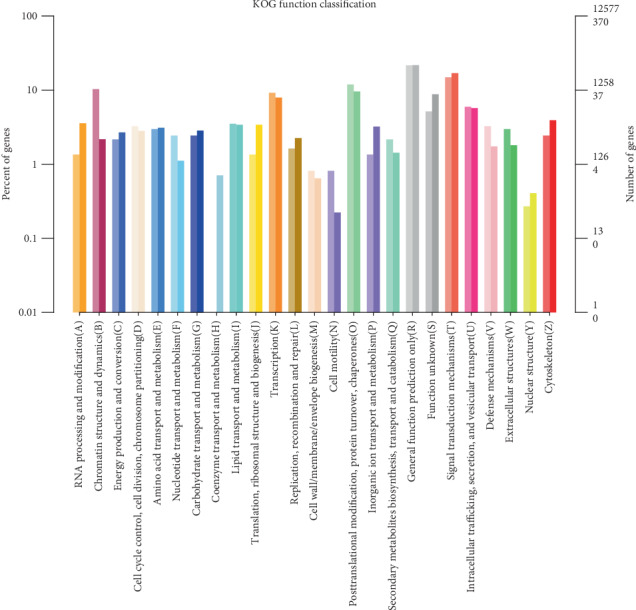
Functional classification of euKaryotic Ortholog Groups (KOG) database on DEGs. The *x*-axis shows the contents of KOG, the left *y*-axis shows the percent of genes, and the right *y*-axis shows the number of genes in the classification.

We also performed KOG annotation to reveal the function distribution of the DEGs to clarify the embodiment of sample differences in gene function. The results of the DEGs classified on the KOG system are shown in Figure 7.

### 3.6. GSEA Identifies a Cisplatin Resistance-Related Signaling Pathway

**Figure 8:**
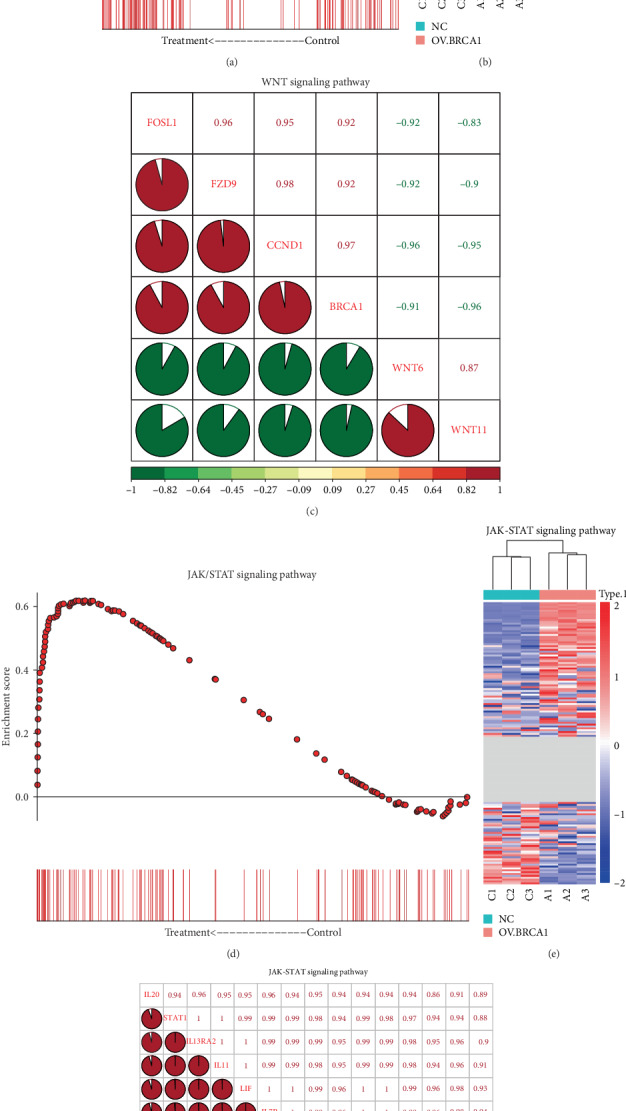
Comprehensive analysis of the WNT and JAK/STAT signaling pathway. (a, d) Enrichment plot of the WNT and JAK/STAT signaling pathway from GSEA. (b, e) The heat map of genes in the WNT and JAK/STAT signaling pathway based on high-throughput sequencing data. (c, f) The relationship between BRCA1 and the key DEGs in the WNT and JAK/STAT signaling pathway.

GSEA results revealed a positive correlation of the DEGs in the OV-BRCA1 group with CCRT resistance-related signaling pathways, such as the WNT signaling pathway and the JAK/STAT signaling pathway (Figures 8(a), 8(b), 8(d), and 8(e)). We intersected the key genes in the WNT and JAK/STAT signaling pathway based on the GSEA results and the DEGs from the BRCA1 overexpression group and obtained 5 key DEGs in the WNT signaling pathway and 14 key DEGs in the JAK/STAT signaling pathway. The correlation analysis indicated a strong positive correlation between BRCA1 and the key DEGs in the WNT and JAK/STAT signaling pathways (Figures 8(c) and 8(f)).

### 3.7. BRCA1 May Affect CCRT Resistance via the JAK/STAT Signaling Pathway

**Figure 9:**
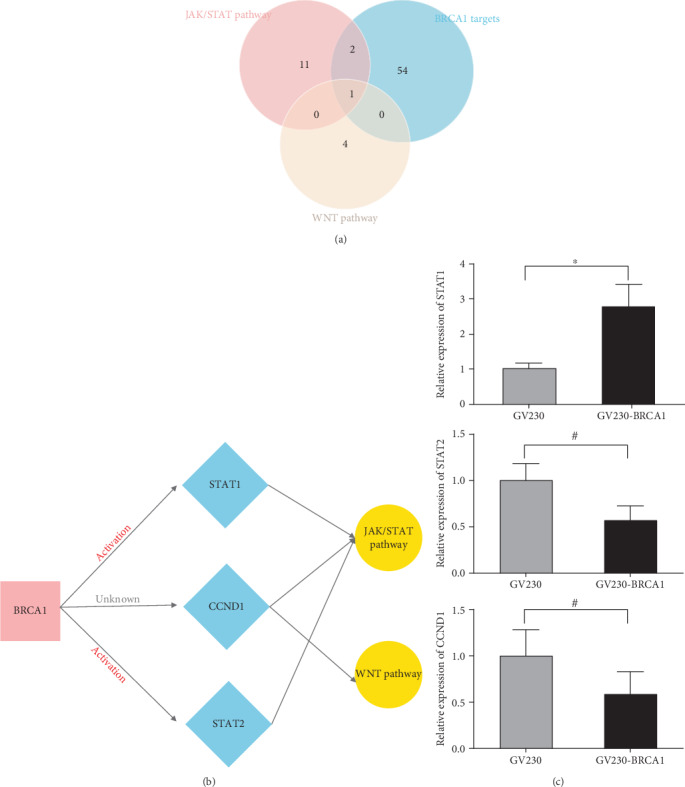
Screening of BRCA1-related CCRT resistance mechanisms in CSCC. (a) The Venn analysis of differentially expressed target genes of BRCA1. (b) The potential regulatory network of BRCA1 in the CCRT resistance of CSCC. (c) The expression of STAT1, STAT2, and CCND1 by qRT-PCR in the SiHa cell with GV230-BRCA1 or GV230 transfection.

To more closely examine the mechanisms of BRCA1 in CCRT resistance in CSCC, we intersected the key genes in the WNT and JAK/STAT signaling pathway and target genes of BRCA1 based on the TRRUST database (Figure 9(a)). We found that BRCA1 may upregulate the expressions of STAT1, STAT2, and CCND1, three key genes in the JAK/STAT and WNT signaling pathway (Figure 9(b)). We performed qPCR verification, and the results showed that only STAT1 was significantly increased in SiHa cells with GV230-BRCA1 plasmid transfection (Figure 9(c)). These findings showed that overexpression of BRCA1 in CSCC cells may enhance resistance to CCRT through activating the JAK/STAT signaling pathway via upregulating STAT1.

## 4. Discussion

CCRT is the standard therapy for locally advanced CSCC; however, the development of intrinsic or acquired CCRT resistance dramatically decreases its clinical effectiveness. To investigate efficient biomarkers that could predict the response to CCRT and serve as potential therapeutic targets for CSCC patients, we performed comprehensive analysis of resistance in CSCC based on a GEO dataset and transcriptome sequencing. First, we reanalyzed the GSE56363 dataset and identified 609 DEGs including 223 upregulated DEGs and 386 downregulated DEGs between NCR and CR patients [18]. We performed GO enrichment for DEGs related to resistance in CSCC and identified 10 hub genes with the highest degrees from the PPI network, including BRCA1, CDCA8, ASPM, CDC45, RAD51, HMMR, CENPF, EXO1, DTL, and ZWINT genes. We next performed transcriptome sequencing of SiHa cells transfected with GV230-BRCA1 or GV230 plasmid and performed functional annotation for DEGs based on GO and KOG database. Finally, we performed GSEA to identify CCRT resistance related signaling pathways to investigate potential molecular mechanisms of resistance in CSCC. STAT1, STAT2, and CCND1 were selected as the differentially expressed target genes of BRCA1 in the WNT and JAK/STAT signaling pathway and may correlate with the CCRT resistance of CSCC.

BRCA1 (BRCA1 DNA repair associated) is a tumor suppressor gene and related to hereditary breast and ovarian cancer. BRCA1 plays a pivotal role in homologous recombination-based DNA repair and functions in both cell cycle checkpoint control and maintenance of genomic stability [30]. Genomic instability caused by DNA repair defect after BRCA1 deletion may lead to tumorigenesis [31, 32]; however, it was also because of DNA repair defect in BRCA1-deficient cells, DNA damage caused by platinum-based chemotherapy or radiation may be substantially enhanced [33–35]. Gao et al. found that advanced or metastatic esophageal squamous cell carcinoma patients with low BRCA1 mRNA expression had increased response rate to cisplatin-based chemotherapy or chemoradiotherapy [36]. For breast cancer and ovarian cancer patients, BRCA1 mutations also increased chemosensitivity and/or radiosensitivity [37, 38]. Our study showed that the expression level of BRCA1 was higher in CSCC resistant to CCRT than that in CSCC sensitive to CCRT by bioinformatics analysis and qPCR verification. ROC curves showed that BRCA1 showed a high diagnostic value in CSCC patients with CR to CCRT, which demonstrated that BRCA1 might be correlated with the sensitivity of CC cells to CCRT.

Through the analysis of our sequencing results, we found that STAT1, STAT2, and CCND1 were upregulated in SiHa cells with BRCA1 overexpression, and these findings might reveal part of the mechanisms by which BRCA1 affects sensitivity to chemoradiotherapy in CSCC. Previous studies found that BRCA1 is required for the upregulation of STAT1 and STAT2, and they play a synergistic role in the differential regulation of IFN-gamma target genes [39, 40]. In breast cancer patients who are BRCA1 mutation carriers, CCND1 expression was significantly reduced [41]. These findings are consistent with our results, demonstrating that BRCA1 expression is positively correlated with STAT1, STAT2, and CCND1. STAT1 and STAT2 are important components of the JAK/STAT pathway, and continued activation of the JAK/STAT signal is associated with tumor progression [42, 43]. CCND1 is often overexpressed in tumors, and its expression is positively correlated with activation of the JAK/STAT pathway [44]. In addition, studies showed the JAK/STAT pathway played a crucial role in cisplatin resistance of CC [45, 46]. Furthermore, our qPCR validation revealed that STAT1 was upregulated in SiHa cells overexpressing BRCA1 in our study. We speculated that BRCA1 may increase the sensitivity of CSCC patients to cisplatin-based CCRT by upregulating STAT1 to activate the JAK/STAT pathway.

## 5. Conclusion

Our work analyzed transcriptome-related changes in CCRT-resistant CSCC patients and SiHa cells based on GEO dataset analysis and transcriptome sequencing. We found that BRCA1 may enhance the sensitivity of CSCC patients to cisplatin-based CCRT by upregulating STAT1 to activate the JAK/STAT pathway. These findings may provide new therapeutic strategies to overcome intrinsic or acquired CCRT resistance in CSCC. Additional studies are needed to further elucidate the specific mechanism of BRCA1 in CCRT resistance of CSCC.

## Figures and Tables

**Table 1:** Primer sequences used for qRT-PCR amplification.

Primer	Sequences
BRCA1	5′-GACAGTCAGCCGCATCTTCT-3′
	5′-ACCAAATCCGTTGACTCCGA-3′
STAT1	5′-ATCAGGCTCAGTCGGGGAATA-3′
	5′-TGGTCTCGTGTTCTCTGTTCT-3′
STAT2	5′-CCAGCTTTACTCGCACAGC-3′
	5′-AGCCTTGGAATCATCACTCCC-3′
CCND1	5′-GCTGCGAAGTGGAAACCATC-3′
	5′-CCTCCTTCTGCACACATTTGAA-3′
GAPDH	5′-GACAGTCAGCCGCATCTTCT-3′
	5′-ACCAAATCCGTTGACTCCGA-3′

**Table 2:** The significantly enriched analysis of DEGs in NCR CSCC patients.

Category	Term	Description	Adjusted *P* value	Count
BP	GO:0009615	Response to virus	2.19*E* − 17	67
BP	GO:0060337	Type I interferon signaling pathway	2.19*E* − 17	35
BP	GO:0071357	Cellular response to type I interferon	2.19*E* − 17	35
BP	GO:0034340	Response to type I interferon	1.36*E* − 16	35
BP	GO:0034341	Response to interferon-gamma	4.28*E* − 16	50
BP	GO:0051607	Defense response to virus	1.64*E* − 15	53
BP	GO:1903900	Regulation of viral life cycle	1.02*E* − 14	40
BP	GO:0071346	Cellular response to interferon-gamma	8.74*E* − 14	44
BP	GO:0060333	Interferon-gamma-mediated signaling pathway	1.01*E* − 13	31
BP	GO:0050792	Regulation of viral process	1.64*E* − 12	42
CC	GO:0071556	Integral component of lumenal side of endoplasmic reticulum membrane	7.23*E* − 06	12
CC	GO:0098553	Lumenal side of endoplasmic reticulum membrane	7.23*E* − 06	12
CC	GO:0005925	Focal adhesion	6.34*E* − 05	49
CC	GO:0005924	Cell-substrate adherens junction	6.34*E* − 05	49
CC	GO:0005788	Endoplasmic reticulum lumen	6.34*E* − 05	40
CC	GO:0030055	Cell-substrate junction	6.90*E* − 05	49
CC	GO:0012507	ER to Golgi transport vesicle membrane	1.29*E* − 04	15
CC	GO:0030176	Integral component of endoplasmic reticulum membrane	2.83*E* − 04	24
CC	GO:0042611	MHC protein complex	3.75*E* − 04	9
CC	GO:0030662	Coated vesicle membrane	3.75*E* − 04	26
MF	GO:0005201	Extracellular matrix structural constituent	8.84*E* − 08	22
MF	GO:0019838	Growth factor binding	9.67*E* − 07	19
MF	GO:0005539	Glycosaminoglycan binding	9.67*E* − 07	24
MF	GO:0005178	Integrin binding	9.67*E* − 07	18
MF	GO:0008201	Heparin binding	4.38*E* − 05	18
MF	GO:0004714	Transmembrane receptor protein tyrosine kinase activity	6.88*E* − 04	10
MF	GO:1901681	Sulfur compound binding	6.88*E* − 04	20
MF	GO:0050839	Cell adhesion molecule binding	1.36*E* − 03	30
MF	GO:0030020	Extracellular matrix structural constituent conferring tensile strength	3.32*E* − 03	7
MF	GO:0019199	Transmembrane receptor protein kinase activity	3.55*E* − 03	10

**Table 3:** The significantly enriched analysis of DEGs between OV-BRCA1 and NC SiHa cells.

Category	Term	Description	Adjusted *P* value	Count
BP	GO:0051607	Defense response to virus	4.34*E* − 25	50
BP	GO:0009615	Response to virus	1.13*E* − 24	57
BP	GO:0060337	Type I interferon signaling pathway	2.42*E* − 19	29
BP	GO:0071357	Cellular response to type I interferon	2.42*E* − 19	29
BP	GO:0034341	Response to interferon-gamma	7.82*E* − 19	40
BP	GO:0071346	Cellular response to interferon-gamma	7.98*E* − 19	38
BP	GO:0034340	Response to type I interferon	7.98*E* − 19	29
BP	GO:0060333	Interferon-gamma-mediated signaling pathway	4.05*E* − 17	27
BP	GO:1903900	Regulation of viral life cycle	1.13*E* − 15	31
BP	GO:0048525	Negative regulation of viral process	8.16*E* − 15	25
CC	GO:0000786	Nucleosome	2.38*E* − 11	31
CC	GO:0044815	DNA packaging complex	1.05*E* − 10	31
CC	GO:0032993	Protein-DNA complex	3.98*E* − 07	36
CC	GO:0071556	Integral component of lumenal side of endoplasmic reticulum membrane	4.23*E* − 06	12
CC	GO:0098553	Lumenal side of endoplasmic reticulum membrane	4.23*E* − 06	12
CC	GO:0005788	Endoplasmic reticulum lumen	4.81*E* − 05	41
CC	GO:0005925	Focal adhesion	8.43*E* − 05	49
CC	GO:0000788	Nuclear nucleosome	8.43*E* − 05	12
CC	GO:0005924	Cell-substrate adherens junction	9.24*E* − 05	49
CC	GO:0030055	Cell-substrate junction	1.10*E* − 04	49
MF	GO:0016763	Transferase activity transferring pentosyl groups	1.26*E* − 03	13
MF	GO:0003950	NAD+ ADP-ribosyltransferase activity	1.26*E* − 03	9
MF	GO:0002020	Protease binding	1.54*E* − 02	20
MF	GO:0005125	Cytokine activity	1.54*E* − 02	29

## Data Availability

The following information was supplied regarding data availability. The transcriptome sequencing data has been uploaded to the GEO database, and the accession number is GSE141558. Expression profiles of GSE56363 in the manuscript were downloaded from NCBI-GEO (https://www.ncbi.nlm.nih.gov/gds).

## Supplementary Materials

Supplementary Materials Table S1: differentially expressed genes in the BRCA1 overexpression group compared with control group. Table S2: summary of sequencing data.
