# Supplementary material for: Effect of BRCA1 on the Concurrent Chemoradiotherapy Resistance of Cervical Squamous Cell Carcinoma Based on Transcriptome Sequencing Analysis
Source: Biomed Res Int. 2020 Jun 21;2020:3598417. doi: 10.1155/2020/3598417 (PMC7333031; doi:10.1155/2020/3598417)
Supplement: Supplementary Materials — Table S1: differentially expressed genes in the BRCA1 overexpression group compared with control group. Table S2: summary of sequencing data. [file 3598417.f1.docx]

Table S1: differentially expressed genes in the BRCA1 overexpression group compared with control group.

| DEGs | Gene symbol |
| --- | --- |
| Downregulated DEGs | TFAP2A, ENO3, TMEM59L, REEP4, THAP8, METTL17, CDK2, BAG1, SURF1, AC099676.1, CCL28, CYR61, SCD, NRM, AFG3L1P, HDGF, HOXA7, COL1A1, SF3A2, LRRC14, SLC22A17, FAM167B, PBXIP1, YBEY, KIF22, TACC3, UBA5, TRIM47, ATP5H, RHOT2, SRSF1, CALM2, PIGZ, PACS2, LZTR1, ACTA2, PROS1, GSTK1, AC011498.8, EIF1, VAMP1, PIH1D1, AC133919.2, PARP6, TSEN54, USP32P3, PPP6R2, ZBED5-AS1, RPS11, CDKN2B, PLEKHH3, NOTUM, KRT17, DGCR6, NAPG, FBXO2, BRICD5, SGK1, AC109326.1, SYT12, NDUFC1, TBC1D9B, RAD51-AS1, NPC2, SRRM2, AL691432.1, AC233723.2, C11orf86, ANKRD9, LRRC61, AC132008.2, HMGXB3, IDI1, PTPA, SLC25A3, ARHGEF1, KAT2A, AKR1B1, RPL13, TMSB4X, AC004656.1, H2AFV, DDB2, AC132812.1, PIEZO1, SHMT1, AC093323.3, ACAP3, KIF18B, CYP27C1, CDK10, ELMOD3, IK, BRAT1, CRISPLD2, AC025259.3, MYO9B, NPIPA5, CDCA3, UBXN6, ARHGAP11B, TYMSOS, RNF208, AC004908.2, MFSD12, AC145207.5, PDK2, ANXA5, ANKLE2, QTRT1, SGSM3, WNT5A, IGFL2-AS1, ZNF76, AC008735.4, CLEC16A, SLF2, AC020916.1, ENKD1, LMNTD2, IL11RA, HSF4, SALL4, THBS1, OSGEPL1, DPP7, PLXNB1, PARP2, NOL3, SLC9A3-AS1, PRC1, AC145207.2, ERMARD, DROSHA, ZBED6CL, CTDSP1, CCNB1, CPOX, LMF2, SSBP3, CENPA, KCNK15, PDLIM4, NDUFA3, MAPK8IP3, NRBP2, UBE2C, WDR34, EHBP1L1, TUBG2, FAM160B2, AC084125.4, GSTP1, TINCR, SLC22A18, ARFGAP2, ETFB, CPSF1, TMSB15A, AC003102.1, POMT1, S100A6, IQGAP3, WASHC2C, RXRB, NDUFB4, SPAG5, TOP2A, WNT11, ZSCAN16-AS1, MLF2, NUB1, CLIP3, SNRNP200, FTH1, LCAT, TMEM80, AL645608.3, THBS3, TIMELESS, APLP1, TUBB3, DRD4, CCNG2, PHF19, AC018755.3, ID3, METTL26, MELTF-AS1, RPL36A-HNRNPH2, TNK2, POP4, SMIM4, YLPM1, DTX3, FDPS, ARNT, UROD, STAG3L5P, ARHGEF17, DBP, AP001542.3, TMA7, PSCA, TELO2, IP6K2, AC048382.2, ATP5G2, MRNIP, IGFL1P1, DYRK1B, BAMBI, TAF1C, ASXL1, TRIP10, M6PR, AP006284.1, AC027020.2, DGCR8, CPSF7, WDR90, PKD1, BTBD2, PHF8, MEGF6, C21orf58, ACTN4, FASN, COG4, TRIM45, KDM1A, RBL2, AL359513.1, TRAPPC6A, CC2D1A, FUK, WFDC21P, INPPL1, TMOD1, KIFC1, CTSF, BHLHE40, KCTD17, NUMA1, BMP1, TMEM14B, MORN4, PILRB, AC016773.1, SPIRE2, ATP13A1, CTSA, KRT8, EPB41L4A, CDC25B, UBAP2L, C19orf53, ZFYVE19, NUP93, CCS, CCNB2, DCBLD1, CCNL2, STXBP1, ABCC3, ILKAP, LAMTOR4, BRD8, MAOB, RGS11, AGFG2, CPT1B, STARD10, CRTC3, ANXA8, PIMREG, AL157392.3, PBX4, CD14, DNMT1, IFT122, CTSK, GTF3C5, AKR1C2, FADS3, RPS10, DDX39B, NAA38, AC110285.2, LENG8, FAM160A2, LAMB2, SNAI3-AS1, MVD, FAM122B, PTMS, CLIC3, MVB12A, LINC01503, CLASRP, MTX2, FAM173B, C16orf59, EMP1, RCE1, CNTROB, OCIAD2, ENG, TMEM30A, CIT, GUSB, GTSE1, CDC20, UGT1A4, STRIP1, GAS2L1, TSC2, AC112484.2, PFN1, UCN, RBM5, CCDC130, CAT, KCNN4, LAMA5, LTBP3, BCAM, EIF3D, HMGN3, RUVBL1, SLC25A29, TRIM29, HR, UBTF, PLA2G4B, CD59, ABCA7, LIG1, EME1, TRAPPC12, MKNK1, TROAP, NUDT9, NPEPL1, ATF7IP2, PCYOX1L, SMPD4, TUBA4A, ATP6V1B1, KLHDC2, TRAFD1, MIA3, CEP164, LIN7B, TPT1P12, C7orf49, MUM1, CAPNS1, USP14, NEAT1, INTS1, PNPLA6, ISY1, PPP1R16A, CDKN1C, AL391069.2, WNT6, DGAT2, AGAP4, BCOR, RACGAP1, GAS2L3, HMGCS1, AL645608.7, TMEM44, PIF1, FOS, SREBF1, NARF, TTC4, TRAPPC6B, ALPP, DAG1, SCRN2, KRT10, DPF2, APOO, CDK2AP1, TUSC2, WHRN, RRM1, RNF123, SNHG11, AL645608.1, CYP24A1, BX284668.5, FBXL6, CDK5RAP1, DAXX, COPS7B, CDK16, PFKL, CCDC137, BLOC1S1, LINC01260, LMNA, NSMF, PPL, SPECC1, PPP1R10, KCNK15-AS1, MRFAP1L1, WDR60, ZDHHC16, CTSD, FTSJ1, VRK1, KMT5C, LY6E, MPDU1, SAMD11, DNAJA4, CFDP1, STX4, ATXN2, NFYC, LFNG, DDX49, NUSAP1, FAM118A, RPS20, NOMO2, SKIL, ZNF300, EHMT2, MAEA, QTRT2, UNC93B1, OXCT1, GLUL, TPD52L1, BAG6, RBP1, KPNA2, RARA, OSBPL7, POLR1D, TBC1D8, STAG3L3, CREB3, GOLGA1, DBI, DDX3X, DNM1L, NPTN, AC026954.2, HIVEP2, R3HDM1, PACSIN3, GIPC1, GRHPR, PAX8-AS1, TUBE1, SNORD14C, BIN1, ATP2A1-AS1, ACADVL, UVRAG, AGPAT1, LRRC8A, GORASP1, SIAE, VKORC1, TMEM184B, CDKN3, MYO18A, TAF6, ZNF692, ALDH16A1, UBE2V1, PRKACB, BAX, TMEM51, CYP26B1, LIMK1, FAIM, EIF4ENIF1, AOX1, FBXL12, HLCS, DNM1, NBPF15, LSM6, PABPC1L, IDH2, BIN3, TCHP, HES6, LIME1, PHLDB1, POLR2B, DHRS7, SMARCE1, GSN, COL6A1, SBNO2, CIRBP, NCOR2, RINT1, AKR1C3, ZNF83, YIPF3, DPAGT1, NUP107, AL132780.3, ACSS2, LINC00116, TSPYL2, ASAH1, RPS3A |
| Up-regulated DEGs | PLS3, CPS1, COPB2, SERPINH1, PNPT1, PLD3, NAA50, SNORD3A, LBHD1, DIABLO, LRRC8D, YY1, PPP2R2A, CEMIP, LRRC75A-AS1, HSPA9, DAZAP1, DUS2, MANBAL, SERPINB2, SP110, HLA-C, PARP14, SCFD1, TWF1, ARPC1B, FN1, PARP12, CANX, KLHDC3, USP7, FEZ2, SAMD9L, PHB, PLOD2, RETSAT, VPS33B, TBCE, ECHDC1, AP3B1, MCCC1, CEACAM20, CCL3L1, VPS29, TCN2, GALNT10, PARP10, G3BP1, U62317.1, LINC02319, AL138885.3, MGAT5, PHACTR4, FURIN, SYNGR2, FAM111A, IFNL1, ZMYM1, RWDD4, NEFL, CDK7, CARD17, AC245748.3, LRRC8B, OSGIN1, SPHK1, CYP27B1, TIGD1, CUL1, PTK2, UGDH, TNFAIP2, RPN1, HRH1, RAI14, TRIM25, CRTC2, DTL, KLF11, SURF4, PDXK, PARP8, RNU6-781P, TBL2, PARP3, UBAC2, IMP3, LGALS9, ZADH2, AGFG1, ABCF1, YTHDF3, SACM1L, PAICS, ZNF266, CDC45, FAS, IFI27, CXCL11, KYAT1, LIMCH1, MPPE1, CDCA7, AC005006.1, AL390719.1, ABCA2, TRIM22, PPFIA1, PPIL2, CXCL10, PRAL, SON, LINC02158, LMAN2L, CEACAM22P, NT5C3A, AL662884.1, POLI, INTS6, ANGPTL4, IRGM, DHRS2, AL109615.3, RMRP, ADSL, AC093001.1, MX1, PIN1, SMUG1, IFIT2, AIM2, IFIT3, APLP2, CCR4, PARP16, CCL20, LINC00161, AC005332.10, KRT34, RN7SL4P, RAP1GAP2, TRAP1, CEACAMP10, SUB1, MX2, KANSL3, AP001189.5, ACSBG1, RN7SL2, EPB41L2, HNRNPL, AC007923.1, MANF, C8orf4, RGMA, CXCL8, RSAD2, LINC01647, MMP1, RYK, LAMP3, DIAPH1, AK4, PMS1, MORF4L1, AC104534.1, NPC1, TEAD2, GRSF1, ZFAND2B, RDH11, ETV7, BATF2, UCHL3, AC002480.1, TRIOBP, LINC01554, TNPO1, RAB11B-AS1, B3GAT3, CD74, MFSD5, ANKLE1, NT5E, SCG5, TSFM, AC139491.2, SRSF9, FBXO36P1, IFI44L, MMP3, RPPH1, GATAD2A, FP236383.3, FP671120.4, FP236383.2, FP671120.3, TAF9, ZFAND2A, ADNP, PGRMC2, ATP2C1, SET, BIRC3, IFIT1, SLC39A1, MAGT1, NRG1, IFI35, ARL14, MXD1, STAT1, PEMT, MRPL19, MFN2, NDUFS7, IDO1, BANF1, BCAP31, IL13RA2, UCK1, DDX60L, IL11, RFC4, DOK4, IL7R, AC012435.1, ECH1, IFI44, RN7SK, LINC01882, OAS2, LDHA, MINK1, EPS8L2, RN7SL5P, RPP14, KLHDC7B, HM13, IL15RA, TBC1D7, GMPR, ISG20, EDC4, TINAGL1, IL10RB, DDX58, INPP1, CD68, WARS, TUBGCP2, CCND1, PPA2, RN7SL1, FAM76B, SLC15A3, CCL5, FAM189B, ZFAND5, HLA-B, OAS1, AL512488.1, INHBA, MIR155HG, CXCL1, SKP1, CD3EAP, KYNU, TMEM267, UBN1, GIT2, RBMS2, CMPK2, GRAMD1B, SAMD9, ATF4, U62317.2, IFIH1, HLA-DRB1, CSTF1, FOSL1, ZCCHC11, YWHAZ, TRIM5, HIST1H2AC, LINC00941, PIAS2, NDUFB9, GBP1, HIST1H3D, AIDA, CASP1, PPP1R21, OASL, ANKRD1, AL645608.8, SRSF4, TNFSF10, ANXA3, PTPRR, RAB31, C1S, CXCL3, GUCD1, AFAP1L1, HCP5, COG8, SERPINE1, DLAT, NMD3, HERC6, ZNF329, GPR87, S100A16, XAF1, HIST1H2BF, CXCL2, NAMPT, XBP1, USP18, SERP1, AL031777.1, TNC, PANK2, RNVU1-7, HLA-DRA, SAA2, HIST1H4H, IFI6, SAR1B, HYOU1, FBXO9, RNA5-8S5, SLC38A9, STC2, AL157871.3, ISG15, LIF, LHX6, U1, RPL26L1, PLEKHA4, RNU1-1, ADAM15, CD44, RCAN1, PTGS2, UBR5, RGS2, DBNDD2, RNVU1-18, AC008736.1, CERCAM, TAP1, NOMO3, CYTOR, RNU1-2, B2M, PMAIP1, IRF7, HLA-F, ARHGAP35, RNU1-3, PDXDC1, TLDC2, DDX60, USP41, FLNB, SMARCAL1, C12orf43, AC004264.2, HES7, PEX5, FST, ZZZ3, SECTM1, SAMHD1, EHD1, PHLDA1, TRIM69, BRMS1L, PCOLCE2, HLA-E, CFB, NDUFAF5, IFITM2, PEAK1, APOL6, HSP90B1, TNFRSF21, ATOX1, AL445490.1, OAS3, UBE2L6, TM4SF1, VEGFC, C15orf48, MIR4435-2HG, PSMB9, KTN1, HMGA1, PGAM1, DTX3L, RELB, STMN3, FJX1, TMEM171, RAET1L, IL6, RNF223, PXK, POLR2E, HIST1H2BG, EIF4E, PLSCR1, OLR1, PARP9, GPBP1L1, EDN1, CPA4, DDIT3, MAPK13, SNORD17, RBM22, NEDD4L, ERAL1, GNL3, GLIPR1, PTPN2, HIST1H2AL, PDE4D, KIAA0040, PPM1K, WDR74, CDCP1, HIST1H1PS1, HERC5, EMC1, IFI16, GAL, HIST1H2BM, GALR2, HLA-A, MDK, KRT81, FKBP11, SLC29A1, P4HB, H1F0, C8orf59, STK26, C1R, PDGFRL, PSMB8, PCSK6, PDIA5, ITPRIP, ZNF644, CARD16, STAT2, PYM1, PRDM2, DAP3, HIST1H1D, MFSD2A, HIST2H4A, ADRB2, CCT5, SAA1, BMP7, PROCR, IDH3A, TMEM141, IFIT5, ARC, MRPS14, RPS19BP1, GDF15, DNAJB9, AC089983.1, CSF1, HIST1H2BJ, CCDC194, CLDN1, UBC, RIPK2, MRPL34, STEAP1, NOCT, SAT1, AK2, RNF216P1, NAMPTP1, UAP1, HIST3H2A, MT-RNR1, CGREF1, SEC61B, IFRD2, ODC1, BORCS8, FAM216A, BCR, RSPO3, TRIM21, G0S2, TMED2, HIST2H4B, MMP23A, PPIL3, ZNF800, RARRES3, RASD1, NUDT16L1, MASTL, HELZ2, ICAM1, IFITM3, MYO1B, VEZT, LGMN, SLCO4A1, DUSP5, AC092368.3, PRKD2, TMEM62, PVR, PLAUR, AL671277.1, GAS5, HIST1H3E, OXR1, SNHG12, HIST3H2BB, UBALD1, SERPINB6, SERPINB9, HSPA8, JAG1, GCA, MGAT1, FABP5, TNFRSF10A, IRAK2, ELF1, NDUFAF2, UBE2E2, ITGB1, VDAC2, DUXAP8, SERPINB1, SEC24D, NLRC5, ERRFI1, NMI, HIST1H2BC, SP100, STC1, EVI2B, CSNK1A1, CIAPIN1, BLZF1, NKX2-8, RNF13, EPCAM, HIST2H3D, HSPA5, AL138724.1, PDIA3P2, RTP3, AP2S1, PSMB10, ZNF330, GADD45A, UBE2E1, Z85994.1, BEX2, UNG, PTX3, DUSP2, CAPRIN2, CKAP4, ACSL3, TNFRSF11B, DMKN, WDR4, AP1G1, EPHA2, C6orf141, BST2, GPAT3, KLF5, CHAF1A, ADM, TXNIP, CTBS, LARP1B, C19orf48, AC010168.2, HIST1H2AE, HIST1H2BL, CRELD2, LAMC2, FANCA, ADAMTS1, SMOC1, RAP1GDS1, STRN4, ZNF92, MLKL, TPM4, AC068768.1, GPR160, TGM2, TRIM14, AC093323.1, MET, IGFBP6, AC124798.1, CDC23, EMC10, IL20, BTC, TDRD7, SLC16A3, DDX27, DNPEP, EIF3E, TMEM158, TMEM154, HIST1H3C, AL021807.1, UBE2QL1, SH2D2A, GLS, HOXD13, AL359915.2, BRCA1, HS6ST2, NUDCD1, TRIM38, MAD1L1, PKM, ZNF766, ZNFX1, PDIA4, APOL3, TRANK1, DNAJC1, AHR, CPT2, FXYD5, DUSP7, HIST1H1E, RIMKLB, SLC16A6, PML, EHD4, LINC00958, AC068152.1, SLC39A6, SLC16A14, AC009948.1, CASP4, IPO5, MRPL55, TMEM173, SLC12A7, METTL1, SNHG8, NFKB2, HIST1H2AJ, AC004817.3, MBLAC2, SLC7A11, ARRDC2, ERLEC1, SOD2, TEX10, AC006262.1, LZTS2, CHPF, CCDC71L, IER3, STX11, EIF4A1, C17orf67, AC116407.2, ZC3HAV1, PPIA, COX4I1, NDEL1, TBC1D2, HK2, ANTXR1, SLC39A8, OAF, ERAP1, ADPGK, HIST1H2BD, LRR1, SH2B3, PANX1, EIF2AK2, AC141586.1, FNTA, C6orf89, FBXO6, SEC24A, FAM84B, LSR, SNORA73B, QSOX1, TAF4B, ZBTB33, OR2B6, HIST2H3C, ZNF670, HIST2H3A, UBA7, MBP, AC016831.1, FOXN2, TNFAIP3, MRPL3, FAM89A, LRRC8C, LRP10, MAL2, ZNF296, CD47, NUCB2, ZNF526, TYMP, AC008035.1, RAB27B, C17orf100, DUS1L, ENTPD7, RNF213, F3, RDH10, CCDC68, RABEPK, LTBR, AC239868.1, TXNDC15, PVT1, PTP4A2, SSR3, EEF1E1, JADE2, SOWAHC, HSD11B2, AP3S1, SQOR, AP002478.1, KITLG, ARHGEF2, FGF2, AC069544.2, TMED5, RICTOR, TMED7-TICAM2, HCG4, HIST1H3G, PPIB, HIST2H2BC, RPLP0, IVNS1ABP, AC145343.1, NCR3LG1, AL049555.1, HIST2H2BE, TIAL1, AMH, ATG12, SOCS1, TSPAN4, ETV4, MPZL1, NR1D1, CPNE7, HIST1H4E, MALAT1, SLC41A2, AC090970.3, BIK, CHST7, PSME2, PHACTR2, FCMR, CDC25A, AGMAT, MRPS18C, FIP1L1, THSD4, CEBPZ, DUSP4, PCGF5, AC016876.2, PPP1R13B, CKB, TSPAN3, BNIP3, CHRNA5, SDE2, FZD9, CTSL, TMEM140, CALR, HIST4H4, HIST1H2AI, APOBEC3F, GRK2, PLK3, WNT7B, ZNF595, ERP29, KCTD1, ADIPOR1, ZIC5, IKBIP, USP8 |

Table S2: Summary of sequencing data

|  | A1 | A3 | A2 | C1 | C2 | C3 |
| --- | --- | --- | --- | --- | --- | --- |
| Total Reads Count(#) | 44838834 | 37385286 | 43701672 | 41257188 | 46637872 | 49169622 |
| Total Bases Count(bp) | 6.73E+09 | 5.61E+09 | 6.56E+09 | 6.19E+09 | 7E+09 | 7.38E+09 |
| Average Read Length(bp) | 150 | 150 | 150 | 150 | 150 | 150 |
| Q10 Bases Count(bp) | 6.73E+09 | 5.61E+09 | 6.56E+09 | 6.19E+09 | 7E+09 | 7.38E+09 |
| Q10 Bases Ratio(%) | 100.00% | 100.00% | 100.00% | 100.00% | 100.00% | 100.00% |
| Q20 Bases Count(bp) | 6.46E+09 | 5.39E+09 | 6.3E+09 | 5.96E+09 | 6.75E+09 | 7.1E+09 |
| Q20 Bases Ratio(%) | 95.99% | 96.18% | 96.18% | 96.30% | 96.43% | 96.30% |
| Q30 Bases Count(bp) | 6.08E+09 | 5.08E+09 | 5.94E+09 | 5.62E+09 | 6.37E+09 | 6.7E+09 |
| Q30 Bases Ratio(%) | 90.40% | 90.61% | 90.68% | 90.80% | 91.07% | 90.83% |
| N Bases Count(bp) | 10230 | 8334 | 9479 | 9125 | 10513 | 10868 |
| N Bases Ratio(%) | 0.00% | 0.00% | 0.00% | 0.00% | 0.00% | 0.00% |
| GC Bases Count(bp) | 3.37E+09 | 2.79E+09 | 3.27E+09 | 3.09E+09 | 3.47E+09 | 3.68E+09 |
| GC Bases Ratio(%) | 50.15% | 49.80% | 49.87% | 49.88% | 49.67% | 49.84% |
